# Fluid-attenuated inversion recovery magnetic resonance imaging textural features as sensitive markers of white matter damage in midlife adults

**DOI:** 10.1093/braincomms/fcac116

**Published:** 2022-05-05

**Authors:** Maria-Eleni Dounavi, Audrey Low, Graciela Muniz-Terrera, Karen Ritchie, Craig W. Ritchie, Li Su, Hugh S. Markus, John T. O’Brien

**Affiliations:** 1 Department of Psychiatry, School of Clinical Medicine, University of Cambridge, Cambridge CB2 0SP, UK; 2 Centre for Dementia Prevention, University of Edinburgh, Edinburgh, UK; 3 Institute for Neurosciences of Montpellier, University of Montpellier, INSERM, Montpellier, France; 4 Department of Neuroscience, University of Sheffield, Sheffield, UK; 5 Department of Clinical Neurosciences, University of Cambridge, Cambridge, UK

**Keywords:** radiomics, small vessel disease, preclinical dementia, textural analysis, white matter hyperintensities

## Abstract

White matter hyperintensities are common radiological findings in ageing and a typical manifestation of cerebral small vessel disease. White matter hyperintensity burden is evaluated by quantifying their volume; however, subtle changes in the white matter may not be captured by white matter hyperintensity volumetry. In this cross-sectional study, we investigated whether magnetic resonance imaging texture of both white matter hyperintensities and normal appearing white matter was associated with reaction time, white matter hyperintensity volume and dementia risk in a midlife cognitively normal population. Data from 183 cognitively healthy midlife adults from the PREVENT-Dementia study (mean age 51.9 ± 5.4; 70% females) were analysed. White matter hyperintensities were segmented from 3 Tesla fluid-attenuated inversion recovery scans using a semi-automated approach. The fluid-attenuated inversion recovery images were bias field corrected and textural features (intensity mean and standard deviation, contrast, energy, entropy, homogeneity) were calculated in white matter hyperintensities and normal appearing white matter based on generated textural maps. Textural features were analysed for associations with white matter hyperintensity volume, reaction time and the Cardiovascular Risk Factors, Aging and Dementia risk score using linear regression models adjusting for age and sex. The extent of normal appearing white matter surrounding white matter hyperintensities demonstrating similar textural associations to white matter hyperintensities was further investigated by defining layers surrounding white matter hyperintensities at increments of 0.86 mm thickness. Lower mean intensity within white matter hyperintensities was a significant predictor of longer reaction time (*t* = −3.77, *P* < 0.01). White matter hyperintensity volume was predicted by textural features within white matter hyperintensities and normal appearing white matter, albeit in opposite directions. A white matter area extending 2.5 – 3.5 mm further from the white matter hyperintensities demonstrated similar associations. White matter hyperintensity volume was not related to reaction time, although interaction analysis revealed that participants with high white matter hyperintensity burden and less homogeneous white matter hyperintensity texture demonstrated slower reaction time. Higher Cardiovascular Risk Factors, Aging, and Dementia score was associated with a heterogeneous normal appearing white matter intensity pattern. Overall, greater homogeneity within white matter hyperintensities and a more heterogeneous normal appearing white matter intensity profile were connected to a higher white matter hyperintensity burden, while heterogeneous intensity was related to prolonged reaction time (white matter hyperintensities of larger volume) and dementia risk (normal appearing white matter). Our results suggest that the quantified textural measures extracted from widely used clinical scans, might capture underlying microstructural damage and might be more sensitive to early pathological changes compared to white matter hyperintensity volumetry.

## Teaser

In healthy midlife adults, Dounavi and Low demonstrate that fluid-attenuated inversion recovery (FLAIR) MRI textural measures emerging from white matter hyperintensities (WMHs) and normal appearing white matter, related to WMH volume, reaction time and dementia risk. The observed associations suggest that FLAIR textural analysis might capture subtle white matter microstructural alterations

## Graphical Abstract

**Graphical Abstract:**
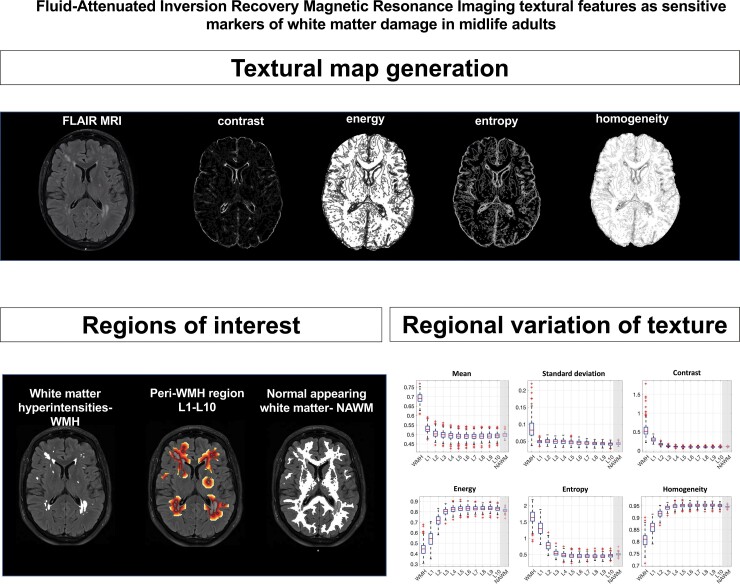

## Introduction

White matter hyperintensities (WMHs) are common radiological findings in the brains of older people, appearing on T_2_-weighted magnetic resonance imaging (MRI), especially fluid-attenuated inversion recovery (FLAIR) scans, which are typically acquired as part of clinical MRI examinations, as patchy areas of increased intensity. WMHs represent microvascular lesions in the brain thought to be caused by localized changes in tissue composition and, although they may be due to several different pathologies, are considered a key indicator of cerebral small vessel disease (SVD).^1^ Importantly, these white matter lesions are associated with poorer cognitive outcomes, incident dementia, stroke and mortality.^2,3^ Furthermore, WMHs are associated with slowed reaction time which is considered as an early feature of SVD^4,5^ and is also a feature of Alzheimer’s disease and mild cognitive impairment.^6^

The WMH burden can be assessed by visual rating scales or by quantifying WMH volume from brain MRI T_2_-weighted or FLAIR images. However, MRI scans have the potential to provide further information about underlying tissue characteristics. Volumetry uses the intensity of every voxel in the image to reach a decision on whether the voxel belongs or does not belong in a particular structure or tissue class (in this case the WMH). A core missed aspect when such approaches are used, has to do with the intensity value of the voxel *per se*. In particular, in each FLAIR scan individual voxel intensities are related to the underlying tissue properties. However, intensity variations within tissue classes are not captured by typical volumetric measurements.

**Figure 1:**
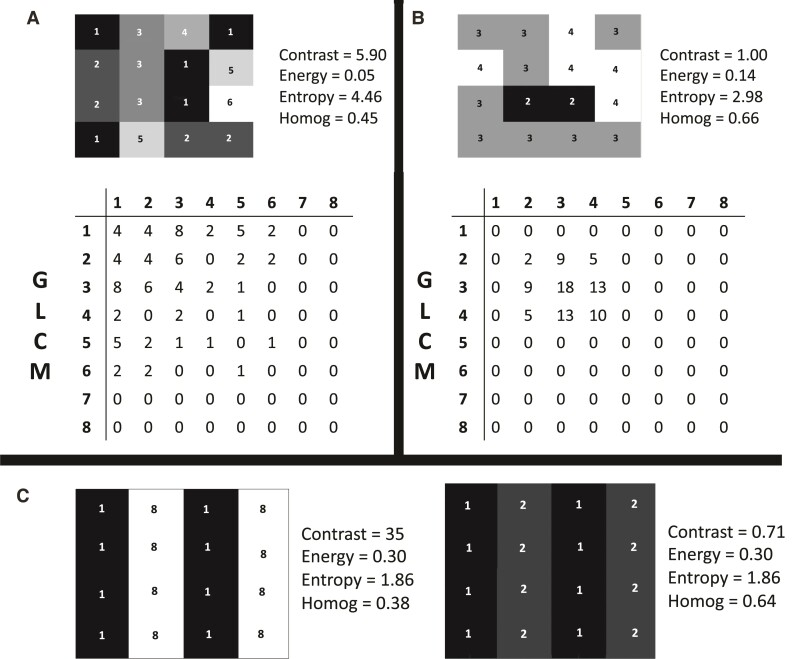
**Grey level co-occurrence matrix (GLCM) generation for example image patches.** Analyses for these patches are run by examining voxel distances of one voxel, eight directions and eight intensity levels. In **A**, a relatively heterogeneous 4 × 4 intensity patch is shown, whereas in **B**, a more homogeneous intensity patch. For case **A**, the values in the matrix are more scattered compared with **B**, where the entries of the matrix are non-zero mainly around the diagonal and for specific intensity pairs. These differences are captured by all quantified textural properties. In **C**, two patches with the exact same entropy and energy are shown, but for which homogeneity and contrast are very different. Homog, homogeneity.

Textural analysis has emerged as a method to provide additional insight on the tissue state, through the analysis of spatial variations in intensity, quantifying properties such as image contrast and homogeneity. Several image textural analysis methods have been proposed in the literature and applied in MRI analysis and are nicely reviewed in Kassner and Thornhill.^7^ Statistical textural features examine spatial relationships of voxel intensities.^8^ One of the most popular methods for textural analysis is the grey level co-occurrence matrix (GLCM) method developed by Haralick *et al*.,^9^ with the generated features belonging to the category of second-order statistical features. Among them, energy (having higher values when there is higher intensity uniformity), entropy (higher entropy is connected with more randomness), homogeneity (higher homogeneity is connected to less differences in intensity) and contrast (higher contrast is connected with larger intensity variations; Fig. 1).^10^

Brain textural analysis has been used to study among others brain tumours,^11^ multiple sclerosis,^12^ stroke^13^ and Alzheimer’s disease^14^ based mainly on T_1_- and T_2_-weighted MRIs. Texture of FLAIR scans has been analysed in relation to blood–brain barrier integrity in stroke patients, where textural homogeneity was increased after administration of gadolinium in patients with increased SVD burden.^13^ In subjects with SVD, textural features predicted conversion to dementia and correlated with cognition.^15^ Textural features have also been shown to differentiate between developing and non-developing normal appearing white matter (NAWM).^16^ Textural analysis has proved to be sensitive in evaluation of the aetiology (ischaemic versus demyelinating) of WMH.^17^ In the context of WMH, textural analysis has also been used to quantify numerous features which are then typically used in a machine learning framework to predict progression of WMH.^16^ Overall, textural analysis has shown sensitivity in detecting damaged tissue and areas or regression/progression in the SVD, multiple sclerosis and brain tumour literature, suggesting that textural features capture underlying tissue damage.

In the present study, our aim was to evaluate whether textural features from FLAIR scans, which were quantified based on a novel approach for textural map generation, were a more sensitive predictor of reaction time compared with WMH volume and the relation of the features to WMH volume and dementia risk. Furthermore, this novel approach allowed us to evaluate the spatial extent of the area surrounding WMH for which the textural features related to reaction time and WMH volume in a manner similar to WMH *per se*. Our overarching aim was to identify textural features relating to WMH pathology and the peri-WMH area demonstrating similar textural patterns to WMH. A small number of comprehensive first-order (mean intensity, standard deviation) and second-order (contrast, energy, entropy and homogeneity) statistical textural features were quantified within WMH and NAWM; the latter ones based on generated textural maps. Our hypotheses were that: (i) textural features would relate to reaction time, (ii) textural features would convey additive information to WMH volume when predicting reaction time, (iii) textural features would be better predictors of WMH burden compared with demographic factors, (iv) a peri-WMH area would demonstrate a distinct textural profile compared with both WMH and NAWM and (v) WMH and NAWM textures (NAWMT) would relate to future dementia risk, such that a more heterogeneous textural pattern would be associated with a higher dementia risk.

## Materials and methods

### Study cohort

Data from the baseline visit of 183 participants from the West London site of the PREVENT-Dementia study were used. PREVENT-Dementia is a longitudinal observational multi-site study in the UK and Ireland.^18^ The protocol of the PREVENT-Dementia study has been described in detail previously.^18^ Cognitively healthy, midlife (age 40–59) participants were recruited through multiple sources. Initially, participants were identified from the dementia register database held at West London Mental Health National Health Service (NHS) Trust, which holds information on patients with dementia and cognitive impairment who have consented to be approached for clinical research and their carers (often offspring). Other participants were recruited via the Join Dementia Research website (https://www.joindementiaresearch.nihr.ac.uk/), or by registering their interest through the PREVENT-Dementia website (https://preventdementia.co.uk/) and public presentations and engagement sessions. The study was approved by the London-Camberwell St Giles National Health Service Ethics Committee (REC reference: 12/LO/1023), which operates according to the Helsinki Declaration of 1975 (and as revised in 1983). All subjects provided written informed consent. The Cardiovascular Risk Factors, Aging and Dementia (CAIDE) risk score incorporating information for age, sex, hypertension, education, activity, body mass index, cholesterol and apolipoprotein ε4 genotype was calculated for all participants.^19^

### MRI protocol

All participants underwent structural MRI acquired on a 3T Siemens Verio scanner. As part of a multi-modal imaging protocol images acquired included three-dimensional T1-weighted MPRAGE [parameters were: 160 slices, repetition time (TR) = 2300 ms, echo time (TE) = 2.98 ms, flip angle = 9°, voxel size = 1 × 1 × 1 mm^3^] and axial FLAIR (parameters were: 27 slices, TR = 9000 ms, TE = 94 ms, flip angle = 150°, voxel size = 0.43 × 0.43 × 4 mm^3^).

### T_1_-weighted image processing

Information from the T_1_-weighted image was used to calculate brain volumes and to retain grey matter (GM) and white matter (WM) maps in the T_1_ space. In particular, estimated total intracranial volume (eTIV), WM and GM volumes were quantified based on the FreeSurfer version 7 pipeline.^20^ The Freesurfer outcome was visually checked and manual corrections were applied in the brainmask or by addition of control points. eTIV was used to normalize the WM, GM as well as the calculated WMH volumes. In the rest of the manuscript when WMH, WM and GM volumes are mentioned, they refer to the normalized values. WM masks in T_1_ space were generated using SPM12 and were subsequently registered to the FLAIR space using FSL FLIRT.^21^

### Quantification of white matter hyperintensity volume

WMH lesion maps were obtained using an automated script on the Statistical Parametric Mapping 8 (SPM8) suite (http://www.fil.ion.ucl.ac.uk/spm/) on FLAIR MRI; details on the procedures involved have been described previously.^22^ T_1_-weighted scans were segmented into GM, WM and cerebrospinal fluid (CSF) based on prior probability maps using SPM8. Brain masks were generated using GM and WM maps, which were used to perform the removal of non-brain matter from FLAIR scans. WMH segmentation was then conducted in FLAIR native space. Initial WMH maps were generated using threshold-based segmentation at a threshold of 1.2 times the median pixel intensity. All WMH maps were reviewed by a single experienced rater blinded to all clinical information, and used as starting points for manual WMH delineation. WMH volumes were normalized by eTIV to account for individual differences in head size [(WMH/eTIV) × 100%] and transformed using cube-root transformation due to skewness.

### Definition of NAWM mask

To investigate how textural properties differ between WMH and NAWM, a NAWM mask was created reflecting tissue without visible WMH. GM, WM and CSF were segmented from the FLAIR scans using SPM12. A WM mask was derived by multiplying the FLAIR-WM mask and the T_1_-weighted WM mask, registered as described in the previous step to FLAIR space to ensure that the WM class did not include any non-WM tissue. Finally, the NAWM mask was obtained by subtracting the WMH from the WM mask and further eroding the image using a 2 × 2 square kernel to limit partial volume effects from GM and CSF.

### Textural analysis

FLAIR images were bias field corrected using Advanced Normalization Tools—ANTs N4.^23^ The brain was extracted from the FLAIR scans using FSL’s brain extraction tool.^24^

Textural analysis of the FLAIR skull-stripped images was conducted using MATLAB R2019b (The MathWorks, Inc., Natick, MA, USA). First-order statistical textural features extracted for WMH and NAWM were the mean (WMHT_mean_, NAWMT_mean_) and standard deviation (WMHT_std_, NAWMT_std_) of the image intensities. These were measured following normalization of the image intensities by subtracting the minimum and dividing with the range of non-zero values present in the image. Second-order statistical textural features were quantified using the GLCM method^9^ and in particular an in-house adaptation of a voxel-wise textural analysis technique proposed by Maani *et al*.^25^ based on the MATLAB built-in functions *graycomatrix* and *graycoprops*.

The GLCM method essentially measures co-occurrence of intensity pairs in multiple directions in an image and constructs an occurrence table which is used for textural feature quantification. In particular, the image is quantized in N levels (N being typically a power of two, for example eight). The algorithm subsequently measures how many times each individual pair of intensities (for example 2–3, 3–8, 1–6) occurs in the image in a number of directions defined by the user of the algorithm (for example eight directions to take into account all eight voxels touching a voxel of interest in a two-dimensional analysis). The distance separating the pixels of interest can be also an input in the algorithm. Subsequently, an N × N table (GLCM matrix) is filled with the number of times each pair occurred. Following this procedure, the GLCM is normalized and textural features are calculated using formulas detailed in the seminal GLCM paper by Haralick *et al*.^9^ A pictorial example of quantized image patches, the GLCM map and calculated textural features is shown in Fig. 1. When the intensity levels within a region are very different between adjacent voxels, the values tend to be higher far from the diagonal of the constructed GLCM, which gives rise to higher contrast. When the intensity is more homogeneous, higher values in the matrix are recorded close to the diagonal. A higher image energy will be given by numbers being higher for a small number of entities. When there is a lot of randomness (entropy), then each table entry tends to have a similar value, meaning that there is not a dominant pattern in the observed intensity combination. Typically, a region of interest (ROI) in an image is selected and the GLCM analysis is run within this region.^26^

**Figure 2:**
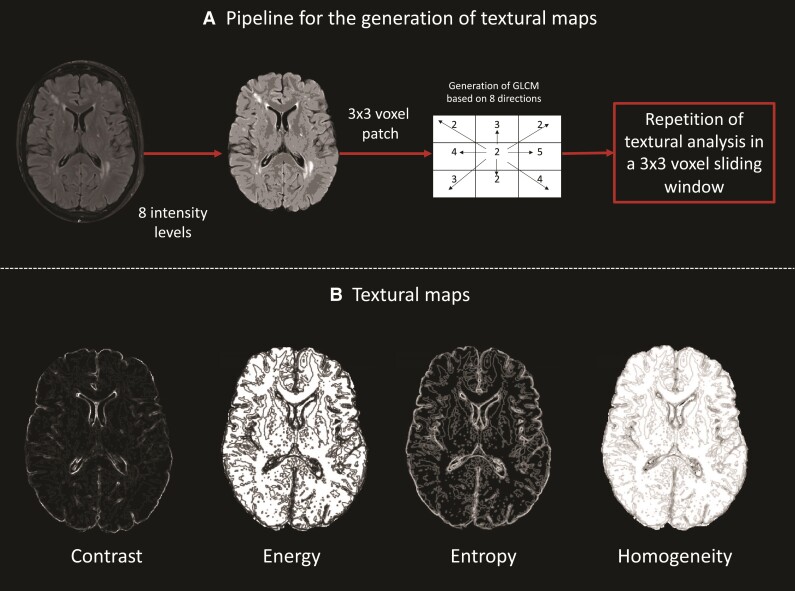
**Pipeline for textural map generation.** (**A**) The bias field corrected FLAIR image is brain extracted and the intensity levels are quantized to eight levels (minimum intensity 1, maximum 8). Subsequently in small 3 × 3 patches the grey level co-occurrence matrices (GLCM) are calculated based on eight directions as shown by the arrows. (**B**) Following that, Haralick features are calculated based on Matlab functions and associated textural maps are generated whereby the intensity of every voxel captures the textural profile of the 3 × 3 voxel neighbourhood centred at every voxel.

The textural analysis pipeline we opted for is an adaptation of the voxel-based GLCM on three orthogonal planes 3D (VGLCM-TOP-3D) technique,^25^ which proposes to run this analysis within a small neighbourhood of voxels in each plane separately (axial in our case). Each voxel is assigned the textural values generated based on its closest neighbours (eight in the present implementation). Hence, this method allows for textural images to be generated. As a result, the extraction of measurements from ROIs can follow the generation of textural maps (Fig. 2) and not vice versa as is customary (i.e. definition of ROIs and application of the textural analysis within the ROI; Supplementary Fig. 1). For our analysis, we have used a quantization level of eight (eight intensity levels in the image), a radius of one voxel surrounding the voxel of interest, thus 3 × 3 voxel analysis patches and eight directions. For every 3 × 3 patch, GLCMs from all eight directions were summed. Haralick features were quantified based on this final GLCM matrix at a voxel-wise level by assigning to each voxel the calculated textural values based on the analysis run in its local 3 × 3 voxel neighbourhood. This procedure is summarized in Fig. 2. Following generation of textural maps, the following textural features were quantified (equations in Supplementary material) within WMH and NAWM: energy (WMHT_energy_, NAWMT_energy_), entropy (WMHT_entropy_, NAWMT_entropy_), homogeneity (WMHT_homog_, NAWMT_homog_) and contrast (WMHT_contrast_, NAWMT_contrast_).

We opted for the generation of textural maps and extraction of mean values rather than running the whole textural analysis pipeline within each individual defined ROI, in order to avoid issues that arise due to ROI selection and GLCM analysis and relate to the maximum and minimum values within the defined ROIs (Supplementary Fig. 1).^27^

All textural measures were transformed using cube-root transformation due to skewness.

### Reaction time

Slowing of behavioural reaction time is a well-documented clinical characteristic of SVD^4^ and Alzheimer's disease.^6^ A simple reaction time task was administered through a touchscreen which records responses and response latencies, as part of the COGNITO battery.^28^ Participants were required to respond by tapping on the screen when a stimulus appeared, and the mean reaction time across 12 successful trials was computed.

### Statistical analysis

To examine differences in textural parameters between WMH tissue and NAWM tissue, paired *t*-tests were used. To test the associations between texture, (i) WMH volume and (ii) reaction time, linear regression models were fitted, adjusting for sex and age. Multiple comparisons were accounted for by using the false discovery rate (FDR) method which was applied to (i) and (ii) separately.^29^ We further added WMH volume*texture as interaction terms to test the interaction between WMH volume and separate textural features in predicting reaction time. We additionally tested whether WMH volumes were associated with reaction time. To examine associations between risk of future dementia (CAIDE score) and MRI textural features at midlife, linear regression models were used. To identify the layers where WM started deviating from the NAWM pattern, we used paired *t*-tests between textural measures in NAWM and the individual layers. Associations of textural features within WMH and NAWM were tested with Spearman correlation. In all regression models, predictors were mean centred. Statistical analyses were conducted using R v4.0 (www.R-project.org/) and MATLAB.

#### Spatial extent of the observed associations

As a further exploratory analysis, we sought to identify the spatial extent of the region surrounding WMH, demonstrating similar textural associations to reaction time and WMH volume with WMHT. For this purpose, we defined 10 layers surrounding the WMH using a two-voxel circular dilation kernel in MATLAB for each axial slice.^30,31^ Thus, WMH maps were dilated using kernels between 2 and 20 voxels with a 2-voxel increment (i.e. 0.86 mm, distance up to 8.6 mm). Each layer mask was multiplied with the WM mask to ensure that non-WM was not included and was exclusive of its previous layer. For each of the identified significant associations from the previous step between WMHT and either reaction time or WMH volume, we have used linear regression models to determine whether the same association persisted in the considered layers using age and sex as additional covariates. FDR was applied for each observed association separately over the 11 ROIs considered.

### Data availability

The data that support the findings of this study are available from the corresponding author, upon reasonable request.

## Results

Sample characteristics are summarized in Table 1.

**Table 1:** Sample characteristics

	Sample (*n* = 183)
Demographics	
Age (years)	51.9 ± 5.4
Sex (% female)	69.9%
Education (years)	16.0 ± 3.4
APOE4 (% carriers)	37.7%
eTIV (cm^3^)	1485.1 ± 150.2
Imaging measures (% of eTIV)	
Total WMH volume	0.11 ± 0.16
Grey matter volume	42.0 ± 2.0
White matter volume	30.5 ± 1.6
Clinical measures	
Reaction time (ms)	341.0 ± 38.5
CAIDE score	5.8 ± 2.9
Diabetes (%)	0.02
Smoking (%)	0.05
Hypertension (%)	14.2

Values are shown as mean ± standard deviation or percentages.

APOE, apolipoprotein; CAIDE, Cardiovascular Risk Factors, Aging and Dementia; eTIV, estimated total intracranial volume; WMH, white matter hyperintensities.

In separate linear regression models, the CAIDE dementia risk score was associated with second-order NAWM textural features but not first-order features: NAWMT_contrast_ (*t* = 4.72, *P* < 0.01, *P*_FDR_ < 0.01), NAWMT_entropy_ (*t* = 4.64, *P* < 0.01, *P*_FDR_ < 0.01) and lower NAWMT_energy_ (*t* = −4.73, *P* < 0.01, *P*_FDR_ < 0.01) and NAWMT_homog_ (*t* = −4.83, *P* < 0.01, *P*_FDR_ < 0.01). Only one WMH textural feature was associated with CAIDE score: WMH_std_ (*t* = 2.32, *P* = 0.02, *P*_FDR_ = 0.05). In a further exploratory model with age, sex, years of education, diabetes, smoking and hypertension status, several associations were observed and are reported in Table 2.

**Table 2:** Associations of textural features in WMH and NAWM with cardiovascular risk factors

	Sex	Age	Educ	Diabetes	Smoking	Hypertension
WMH texture						
Mean	−0.75; 0.45	0.67; 0.51	−0.25; 0.81	−0.70; 0.48	0.24; 0.81	0.08; 0.93
Standard deviation	0.97; 0.33	1.60; 0.11	−0.17; 0.86	0.03; 0.98	1.04; 0.30	**2.18; 0.03**
Contrast	2.44; 0.02*	0.43; 0.67	−0.64; 0.52	−0.12; 0.91	0.47; 0.64	1.74; 0.08
Energy	−2.51; 0.01*	1.61; 0.11	−0.06; 0.96	−0.55; 0.58	0.15; 0.88	0.70; 0.49
Entropy	2.47; 0.01*	−1.66; 0.10	0.04; 0.97	0.76; 0.45	−0.24; 0.81	−0.88; 0.38
Homogeneity	−2.43; 0.02*	1.20; 0.23	0.47; 0.64	−0.30; 0.77	0.20; 0.84	0.14; 0.89
NAWM texture						
Mean	0.23; 0.82	0.25; 0.80	−0.96; 0.34	−0.99; 0.32	−0.27; 0.79	0.52; 0.61
Standard deviation	−2.60; 0.01*	−2.41; 0.02*	1.63; 0.11	1.77; 0.08	0.16; 0.87	−0.97; 0.33
Contrast	−5.11; <0.01*	3.17; <0.01*	−0.83; 0.41	0.02; 0.98	0.63; 0.53	1.33; 0.18
Energy	4.31; <0.01*	−3.04; <0.01*	0.57; 0.57	0.07; 0.95	−0.50; 0.62	−1.18; 0.24
Entropy	−4.27; <0.01*	2.95; <0.01*	−0.53; 0.59	−0.06; 0.95	0.54; 0.59	1.14; 0.26
Homogeneity	5.15; <0.01*	−3.28; <0.01*	0.87; 0.38	−0.01; 0.99	−0.61; 0.54	−1.39; 0.17

The values are presented as *t*-value; *P*-value, resulting from the applied linear regression models. Positive sign in sex indicates higher values in females, in diabetes higher values in diabetes patients, in smoking higher values in smokers and in hypertension, higher values in hypertensive patients. Bold is used to indicate significant findings at a level of *P* < 0.05 and asterisks indicate findings surviving FDR at a level of 0.05.

### Textural differences between WMH and NAWM

**Figure 3:**
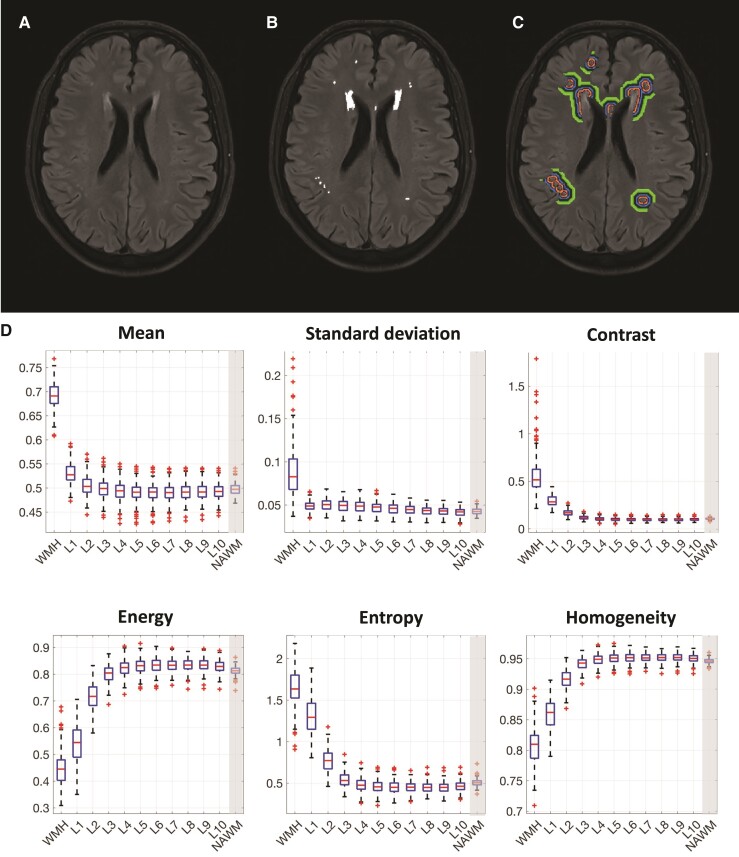
**Variation of texture properties with an increasing radius extending from the WMH *per se* to 10 layers surrounding the WMH (two-voxel dilation kernel).** (**A**) Raw FLAIR image. (**B**) WMH lesion maps were generated based on a semi-automated pipeline. (**C**) Ten layers surrounding the WMH based on a two-voxel dilation kernel and confined within normal appearing white matter. (**D**) Textural values within the whole NAWM are shown in the last column of the boxplots as a reference. Boxplots (horizontal lines within the boxes correspond to the median, the upper and lower ends of the boxes to the 25th and 75 percentiles, crosses indicate outliers and whiskers cover the range of data points not considered outliers) are based on the raw textural values from the 183 participants. NAWM, normal appearing white matter; WMH, white matter hyperintensities.

Compared with NAWM, WMH demonstrated a pattern of higher mean intensity, higher standard deviation, higher contrast, higher entropy, lower energy and lower homogeneity (*P* < 0.001). Examination of textural properties within the 10 defined layers revealed a distinctive change in first-order textural features (i.e. mean and standard deviation) between the boundaries of WMH and the first layer of NAWM (i.e. layer closest to the WMH), while changes in second-order textural features (contrast, energy, entropy and homogeneity) demonstrated graduated changes moving from WMH to NAWM (Fig. 3). Paired *t*-tests between WMH textural features and texture within the layers revealed that the textural profile of each layer was different to WMH texture. The same association was observed for texture within the layers and texture within the whole NAWM. Associations between textural features within WMH and NAWM were examined with Spearman correlations (Supplementary Fig. 2). Within WMH, WMHT_mean_ and WMHT_std_ were moderately associated, WMHT_energy_, WMHT_homog_ and WMHT_entropy_ were strongly associated between them and moderately associated with WMHT_contrast_ and first- and second-order features were weakly moderately associated. Within NAWM, first- and second-order features were not associated. NAWMT_mean_ and NAWMT_std_ were moderately associated and second-order textural features were perfectly associated (Supplementary Fig. 2), with this difference between WMHT and NAWMT textural associations likely related to the extent of the considered regions.

### Associations between texture and WMH volume

General linear models adjusting for sex and age showed that total WMH volume was associated with higher WMHT_std_, higher WMHT_energy_, lower WMHT_entropy_ and greater WMHT_homog_ as well as higher NAWMT_contrast_, lower NAWMT_energy_, higher NAWMT_entropy_ and lower NAWMT_homog_ (Table 3).

**Table 3:** Association between WMH volume and textural properties

	*t*-value	*P*-value
WMH texture		
Mean	1.75	0.08
Standard deviation	5.48	<0.01*
Contrast	−0.41	0.68
Energy	6.60	<0.01*
Entropy	−7.22	<0.01*
Homogeneity	5.30	<0.01*
NAWM texture		
Mean	0.74	0.46
Standard deviation	−1.89	0.06
Contrast	2.90	<0.01*
Energy	−2.79	0.01*
Entropy	2.71	0.01*
Homogeneity	−3.01	<0.01*

*t*-statistic and *P*-values for the conducted linear regression analyses.

WMH, white matter hyperintensities; NAWM, normal appearing white matter .

* Survive FDR correction at a level of *P* < 0.05.

### Association between textural features and reaction time

In a general linear model adjusting for sex and age, WMH volume was not associated with reaction time. Among textural features, only WMHT_mean_ was significantly related to reaction time (*t* = −3.77, *P* < 0.01, *P*_FDR_ < 0.01), whereby higher mean intensities were related to lower reaction time. This association remained significant with the addition of WMH volume, diabetes, smoking and hypertension as a further covariates (*t* = −3.79, *P* < 0.01).

**Figure 4:**
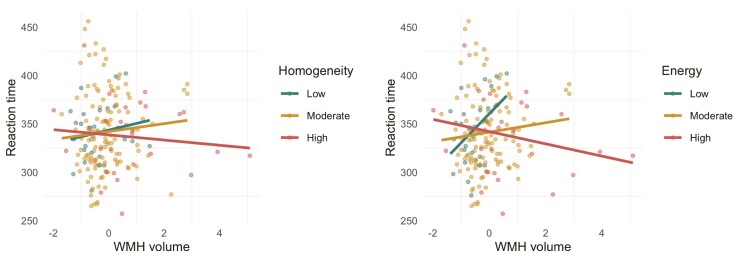
**Plot of estimated marginal means of reaction time depicts a significant interaction between white matter hyperintensity (WMH) volume and texture on reaction time for the 183 participants.** Higher reaction time (in milliseconds) indicates poorer performance. In legend, moderate represents mean homogeneity/energy, while high and low homogeneity/energy was defined as ±1 SD from mean. WMH volume as a percentage of total intracranial volume was cube root transformed for normality.

Interaction analysis in general linear models adjusting for sex and age, revealed that total WMH volume interacted with WMHT_energy_ (*t* = −2.06, *P* = 0.04, *P*_FDR_ = 0.21) and WMHT_homog_ (*t* = −2.06, *P* = .04, *P*_FDR_ = 0.21) to predict reaction time, whereby greater WMH volume was related to prolonged reaction time in cases of low WMHT_energy_ and WMHT_homog_ (Fig. 4).

### Extent of area demonstrating similar textural associations to WMH

**Figure 5:**
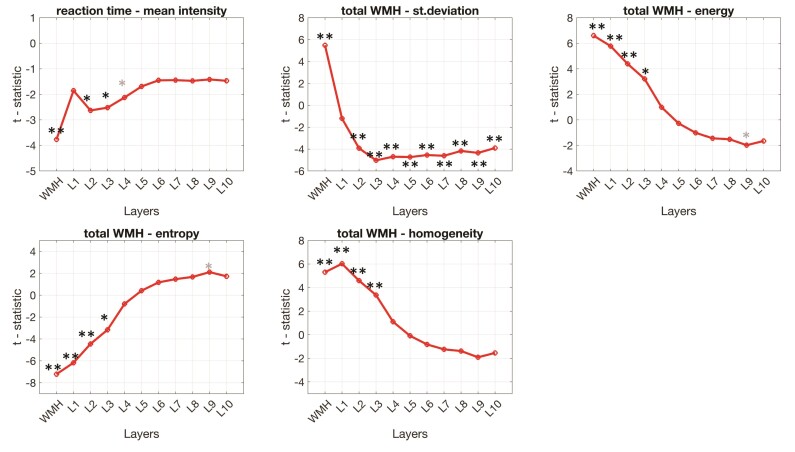
**Extent of the area surrounding WMH where the observed relationships between reaction time and texture, and WMH volume and texture persisted.** For the majority of the examined metrics, the association persisted until layer 3, which corresponds to an area of around 2.6 mm surrounding the WMH. The association between reaction time and mean intensity persisted until Layer 4 (3.44 mm). Plots are based on the full sample of 183 individuals and demonstrate the *t*-statistic from linear regression models with age and sex as additional predictors in the *y*-axis, the layer number on the *x*-axis and asterisks depict the level of significance of the observed association—if any on the respective data points. * *P* < 0.05; ** *P* < 0.01. Dark asterisks indicate associations that survived FDR correction, whereas light asterisks are used for associations that did not survive the correction for multiple comparisons.

For the identified effects, we further investigated the extent of the area immediately surrounding the WMH demonstrating similar textural associations with reaction time or total WMH volume as WMHT, by examining the same association within the 10 defined layers. Results are shown in Fig. 5. In this exploratory analysis, we found that mean intensity was associated negatively to reaction time, up to an area extending until Layer 4 (3.44 mm). Standard deviation of WMH was positively associated with WMH volume. Extending further from the WMH (distance >0.86 mm), the association turned negative for the majority of the layers. Energy, entropy and homogeneity remained significantly associated with the WMH volume until Layer 3 (2.58 mm). The pattern of association between WMH volume and WMHT_energy_, WMHT_entropy_ and WMHT_homog_ reverted after layer 5 (4.3 mm).

## Discussion

Texture analysis as a means to capture spatial patterns of intensity variations allows to capitalize on the fact that the MRI pixel intensity is a reflection of the underlying tissue properties. In the present study, we have shown that in this midlife cohort with a low WMH burden, textural properties of both WMH and NAWM were associated with reaction time (mean intensity), dementia risk and WMH burden (standard deviation, second-order textural features) and interacted with WMH volume to predict reaction time (energy, homogeneity). We further demonstrated the potential of textural analysis of FLAIR images to capture early patterns of textural alterations in a peri-WMH area. Hence, as hypothesized, textural features confer additive information over WMH volume and might have the potential to be used as markers of WM damage.

Reaction time, a cognitive domain known to be influenced in SVD and Alzheimer's disease, was not associated with WMH volume; however, it was associated with WMHT_mean_. The lack of direct associations between WMH volume and reaction time may be due to the relatively low cerebrovascular burden in this healthy midlife cohort.^32^ Despite this, textural features demonstrated significant associations with reaction time, suggesting that FLAIR texture may be capturing additional information and might be more sensitive compared with WMH volume. The finding that slower reaction time was connected to more hypointense WMH was a counterintuitive one. In a study evaluating an intensity-based metric of WM damage, WMHs of lower intensity were associated with more pronounced WMH progression as defined based on WMH volumetry in stroke patients.^33^ A potential explanation could be that a higher mean intensity is associated with newer WMH, whereas a lower WMH intensity is associated with long-standing WMH. For instance, brighter lesions in multiple sclerosis have been thought to reflect more recent events and potentially active inflammation with the temporal evolution of lesion intensity being viewed as a possible marker of reparative capacity.^34^ In particular, longitudinal hyperintense signal reductions are thought to reflect tissue reparative efforts/remyelination.^35^ A longitudinal study of the evolution of the textural properties of the examined lesions and changes in performance in the reaction time task would thus shed further light into the association between WMHT_mean_ and reaction time.

Although unrelated to reaction time when considered independently, interactions of textural measures with WMH volume revealed that a higher WMH volume, when accompanied by lower WMHT_homog_ and lower WMHT_energy_, was associated with higher reaction time. In a post-mortem study of multiple sclerosis patients, it was found that decreased lesion textural homogeneity was associated with completely demyelinated lesions.^36^ In another study, a combination of WMH features extracted from T_1_-weighted images (among them WMH volume, contrast and lesion position) was used to classify individual into different classes capturing distinct WMH severity.^37^ In this latter study, the class with the higher WMH burden comprised participants who were older, with higher blood pressure, higher Framingham risk score and were less active. WMH within that class were less myelinated (T_1_/T_2_ mapping) with relatively high contrast,^37^ although it needs to be mentioned that the notion that the T_1_/T_2_ ratio is a good proxy for myelination has been challenged.^38^ Taken together, characteristics of the intensity profile of WMH might be defining how WMHs impact reaction time, especially in young or middle-aged cohorts with a relatively low WMH burden, where the effect of WMH, as captured by WMH volumetry is not yet prominent in cognition.

The WMH volume was associated with several textural features of both WMH and NAWM, although notably in opposite directions, such that higher WMH volumes were observed in subjects with more homogeneous WMH and less homogeneous NAWM textural profiles. WMHT_std_, WMHT_energy_ and WMHT_homog_ were positively associated with WMH volume, whereas WMHT_entropy_ had a negative association. On the contrary, NAWMT_entropy_ and NAWMT_contrast_ were positively associated with WMH volume, whereas NAWMT_energy_ and NAWMT_homog_ were negatively associated. A potential explanation is that a higher WMH volume is related to more recent ischaemic events which would explain the higher homogeneity as explained previously. Further to that though, more homogeneous WMH concomitant with higher volume could relate to more developed or severe WM damage. Our finding of increased textural homogeneity with a higher WMH volume is in line with previous reports of positive associations between the Fazekas score and textural homogeneity within WMH.^13^ More heterogeneous NAWMT could potentially allude to microstructural alterations happening in NAWM, which could relate to a more severe WMH burden, or increased prevalence of other cerebrovascular pathologies in individuals with higher WMH volume. Associations between textural properties, WMH volume, reaction time and CAIDE may imply that texture indirectly measures WMH severity more accurately than WMH segmentation and volumetry on FLAIR MRI, or that textural measures may be measuring microstructural changes beyond WMH. The negative association between NAWMT_std_ and WMH volume persisted for all the examined layers extending from the WMH apart from the closest layer (0.86 mm from the WMH).

As a further subanalysis, we examined the extent of the area surrounding WMH that demonstrated a similar textural profile to that of WMHT, by analysing incremental layers of NAWM in which textural associations resembled associations observed in WMH. We have shown that an area of around 3.44 mm surrounding the WMH and classified as NAWM demonstrates similar textural associations to the volume of WMH and reaction time as WMHT.

The spatial extent of the peri-WMH area is similar to the extent of penumbras determined in studies using less readily available MRI sequences such as diffusion tensor imaging (DTI; 2–10 mm),^39^ although studies using arterial spin labelling have identified larger penumbras (7–10 mm).^30^ Hence, our technique might demonstrate sensitivity similar to that of DTI in the definition of WMH penumbras, though a direct comparison of the sensitivity of the techniques has not yet been made. The clinical implication of this is that textural properties from conventional FLAIR images obtained in clinical MRI examinations may be sufficiently sensitive to microstructural changes that are undetectable with the human eye and are not captured by volumetry. While the advantage of using FLAIR over DTI lies in its availability, DTI metrics have the advantage of being adjusted for the influence of free water, which cannot be done in FLAIR at present. Similar efforts to generate meaningful measures of WM damage utilizing image intensity information have been conducted in the past. In particular, it has been shown that a metric quantifying relative intensity differences between WMH and NAWM was more associated with visual rating scales compared with WMH volume.^33^

We have further investigated how the CAIDE score, capturing genetic and lifestyle risk factors for dementia was related to textural features. CAIDE was associated with a heterogeneous intensity pattern in NAWM, a finding which further supports the hypothesis that WM textural analysis might be capturing subtle microstructural alterations in clinical scans. In this same cohort, previous analysis using the T_1_-weighted images suggested limited areas of atrophy in subjects with a higher CAIDE.^40^ In the past, it has been shown that entropy and contrast of T_1_ images relate to tau burden in the neocortex.^41^ A further analysis with cardiovascular risk factors, age and sex as predictors unveiled that females had a different textural profile compared with males in both WMH (more heterogeneous textural profile) and NAWM (less heterogeneous), with ageing mainly related to textural alterations in NAWM (more heterogeneous). From the considered cardiovascular risk factors only hypertension was related to a higher WMHT_std_, a finding which did not remain significant following FDR correction.

Overall, we have shown that textural features extracted from images typically used in clinical settings can reveal further information pertaining to damage of WM above and beyond that captured by the volume of WMH. We propose that intensity information from the FLAIR scans holds additional clinical value and could be considered as a marker of WMH severity. It is worth noting that the running time of textural analysis for the FLAIR scans was approximately 8 minutes per subject.

Strengths of our study include generation of textural maps and subsequent extraction of textural values from defined ROIs, rather than running a separate textural analysis within each ROI which renders the quantized intensity values dependent on ROI definition. To achieve this, we have extrapolated a method developed for texture-based morphometry in T_1_-weighted images and applied it in FLAIR space using a two-dimensional approach. This allowed for the quantized intensity levels to be stable across our analysis. Absence of associations between WMH volume and reaction time allowed us to evaluate the sensitivity of the employed technique to detect potential subtle underlying damage. The advantage of investigating cognitively healthy midlife adults stems from the ability to detect early preclinical changes years before the onset of dementia, allowing us to identify earlier and more sensitive predictors of future cognitive impairment. On the other hand, results obtained in our midlife cohort may not extend to elderly cohorts, and future replication in older samples will be needed. Other limitations of this study relate to its cross-sectional nature, which does not allow us to assess the sensitivity of textural parameters in WMH progression. In addition, a single MRI modality was used and sequences such as T_2_ relaxometry, which are sensitive in capturing microstructural damage^42^ were not included in the protocol. The applied normalization step for the first-order textural features does not correct for potential acquisition-related intensity variations. In addition, the confounding effect of other SVD pathologies was not considered in this study. Furthermore, we chose to focus on a limited number of well-defined, easily perceived textural features; in the future, a further set of textural features (statistical and spectral) could be considered.

In conclusion, we have shown that textural properties of FLAIR images are associated with reaction time in a midlife cohort, while WMH volume was not associated with it. Textural properties of WMH interacted with WMH volume to predict reaction time, revealing that a less homogeneous intensity profile associates with worse performance in the reaction time task. Future dementia risk was also associated with NAWM textural properties. Thus, textural features could potentially convey valuable clinical information in terms of the severity of WMH and could be a sensitive measure of SVD. This could imply that limitations associated with the one-dimensional approach of using WMH volume as a measure of investigating WMH pathology can be partly circumvented by incorporating textural features in the analysis.

## Supplementary Material

fcac116_Supplementary_Data

## Abbreviations

CAIDE =: Cardiovascular Risk Factors, Aging and Dementia
CSF =: cerebrospinal fluid
eTIV =: estimated total intracranial volume
FLAIR =: fluid-attenuated inversion recovery
GLCM =: grey level co-occurrence matrix
GM =: grey matter
NAWMT =: normal appearing white matter texture
ROI =: region of interest
SVD =: small vessel disease
TE =: echo time
TR =: repetition time
WM =: white matter
WMHT =: white matter hyperintensities texture
