# Supplementary material for: Fluid-attenuated inversion recovery magnetic resonance imaging textural features as sensitive markers of white matter damage in midlife adults
Source: Brain Commun. 2022 May 5;4(3):fcac116. doi: 10.1093/braincomms/fcac116 (PMC9123845; doi:10.1093/braincomms/fcac116)
Supplement: fcac116_Supplementary_Data [file fcac116_supplementary_data.docx]

**Supplementary material**

Equations used to calculate second order textural features based on Haralick et al. (Haralick, Shanmugam, and Dinstein 1973), where i and j are used to index the GLCM matrix and p(i,j) is the values of the GLCM matrix in the position [i,j].

$$Contrast= \sum_{i} \sum_{j} {|i-j|}^{2} p(i,j)$$

$$Energy= \sum_{i} \sum_{j} p^{2}(i,j)$$

$$Entropy=-\sum_{i} \sum_{j} p\left( i,j \right)\log(p(i,j))$$

$$Homogeneity=\sum_{i} \sum_{j} \frac{p(i,j)}{1+|i-j|}$$

**Supplementary figures**

**Supplementary figure 1**

Supplementary Figure 1: **Conventional approach for ROI-based textural analysis**. For each FLAIR slice (i), regions of interest (ROI) are drawn to demonstrate the variation of the features based on the intensities present within the area using the ROI-based approach. Five ROIs of 26x26 voxels each were defined to demonstrate this (ii). This ROI-based approach is typically used in studies, whereby intensity quantization takes place within an ROI (e.g., 8 intensity levels) and textural analysis follows (iii). On the contrary, when generating textural maps the whole image is quantized to for example 8 levels (iv). Values for the quantified textural features within these ROIs are shown in the accompanying tables. In the ‘Texture using ROI first’ table, the values are shown for typically employed analysis. In the ‘Texture using maps’ table values are shown for the textural map generation analysis approach we opted for. As seen this approach gives more meaningful values in areas where intensity is visibly homogeneous (region 1).

**Supplementary figure 2**

Supplementary Figure 2: **Correlations between textural features within white matter hyperintensities (WMH) and normal appearing white matter (NAWM).** Analysis was conducted using Spearman correlations. Values in red indicate significant associations. Correlation coefficients are reported. Abbreviations: std = standard deviation; cont = contrast; enrg = energy; entr = entropy; homog = homogeneity

**References**

Haralick, R. M., K. Shanmugam, and I. Dinstein. 1973. 'Textural Features for Image Classification', *IEEE Transactions on Systems, Man, and Cybernetics*, SMC-3: 610-21.
